# MEEhubs2024: A hub-based conference on microbial ecology and evolution fostering sustainability

**DOI:** 10.1093/femsle/fnaf022

**Published:** 2025-02-06

**Authors:** Ariane Wenger, Erik Bakkeren, Elisa Granato, Robin Tecon, Sara Mitri, Wolfram Möbius

**Affiliations:** ETH Zurich, Department of Environmental Systems Science TdLab, Zurich 8092, Switzerland; University of Oxford, Department of Biology, Oxford OX1 3RB, United Kingdom; University of Oxford, Department of Biochemistry, Oxford OX1 3QU, United Kingdom; University of Oxford, Department of Biology, Oxford OX1 3RB, United Kingdom; University of Oxford, Department of Biochemistry, Oxford OX1 3QU, United Kingdom; University of Lausanne, Department of Fundamental Microbiology, Lausanne 1015, Switzerland; University of Lausanne, Department of Fundamental Microbiology, Lausanne 1015, Switzerland; University of Exeter, Living Systems Institute, Faculty of Health and Life Sciences, Exeter EX4 4QD, United Kingdom; University of Exeter, Physics and Astronomy, Faculty of Environment, Science and Economy, Exeter EX4 4QL, United Kingdom

**Keywords:** scientific conference, sustainability, inclusivity, hybrid conference, microbial ecology, microbial evolution

## Abstract

Scientific conferences are essential to academic exchange. However, related air travel contributes to greenhouse gas emissions, while expensive registration and travel costs limit the participation of early career researchers and those from low-income countries. Virtual conferences offer promising solutions for reducing emissions and enhancing accessibility and inclusivity but often limit networking and personal interaction. Hybrid multi-hub conferences, which combine virtually connected in-person venues with individual virtual participation, combine the benefits of both conference formats. Thus, we present and discuss MEEhubs2024, a multi-hub conference on microbial ecology and evolution held in January 2024. During this 3-day conference, attendees participated virtually or at one of six hubs in Europe and North America. We analyzed the participants’ and organizers’ feedback to create a template and provide insights into the scientific community's adoption of this new conference format, which was positively evaluated by most participants. Because technical, logistical, and structural challenges remain, including limited opportunities to interact and network across hubs and participation modes, we provide recommendations for improvement, such as hiring technical hosts and offering virtual-only social activities. Finally, we used the participants’ feedback to reflect on conference expectations, highlighting research gaps and the need for organizers to define and communicate goals when organizing conferences.

## Teaser

This paper presents a multi-hub conference on microbial ecology and evolution, analyzing participant and organizer feedback to provide a template and recommendations for future sustainable conferences.

## Introduction

Attending scientific conferences is an integral part of a successful academic career (Oester et al. 2017). These events serve not only as important opportunities for researchers to exchange and disseminate scientific knowledge and advancements but also to build, maintain, and expand their professional network. Furthermore, conferences provide space to gain visibility in the academic community and to develop joint collaborations and projects, thereby advancing academic careers (Edelheim et al. 2018, Hauss 2021).

Nevertheless, conferences exacerbate social inequalities and, due mainly to air travel, contribute significantly to greenhouse gas (GHG) emissions (Cohen and Gössling 2015, Klöwer et al. 2020, Skiles et al. 2021). In response, there have been calls to decarbonize research practices through more sustainable conferences (Wynes et al. 2019, Neugebauer et al. 2020). Since the onset of the COVID-19 pandemic, virtual conferences have become familiar alternatives for decreasing the carbon footprint of conferences by up to 90% (Tao et al. 2021). Virtual conferences also increase accessibility and inclusivity for less privileged researchers, thereby expanding the diversity of conference attendees in terms of gender, career stage, and geographic location (Reshef et al. 2020, Huyck et al. 2021, Skiles et al. 2021). Additional benefits of virtual conferences include lower costs, flexible participation opportunities, and the avoidance of jet lag and travel-related stress (Fraser et al. 2017, Counsell et al. 2020, Foramitti et al. 2021).

Despite these advantages, virtual conferences have challenges, such as technical hiccups, Zoom fatigue, data security issues, and time zone issues (Schwarz et al. 2020, Foramitti et al. 2021, Moss et al. 2021). The biggest drawback is the perceived lack of opportunities for interpersonal connection and building collaborations (Roos et al. 2020, Foramitti et al. 2021); specifically, the spontaneous and casual discussions usually held during meals and coffee breaks are perceived as missing from virtual conferences (Seidenberg et al. 2021, van de Glind and Gomez‐Baggethun 2022). However, a recent study found that when organizers thoughtfully select appropriate technical tools and design intentional, dedicated networking sessions, virtual conferences can achieve effective networking (Wenger 2023).

Hybrid conferences that combine in-person and virtual attendance aim to address the shortcomings of virtual conferences while retaining their benefits, although attendees may perceive and experience the success of these efforts in different ways. Hybrid multi-hub conferences held at several in-person venues that facilitate individual virtual participation through virtual connection create opportunities to maintain in-person interactions while increasing accessibility and decreasing GHG emissions (Reshef et al. 2020, Tao et al. 2021, Moss et al. 2022).

The hybrid conference format is novel, having gained prominence only since the slow repeal of COVID-19 travel restrictions. The hybrid multi-hub conference format, specifically, has not often been implemented in practice. Few studies on multi-hub conferences exist, and these mainly focus on conference logistics (Reshef et al. 2020, Kremser et al. 2023). Thus, the literature lacks a perspective that combines the experiences of both conference organizers and attendees of hybrid multi-hub conferences.

**Figure 1.**
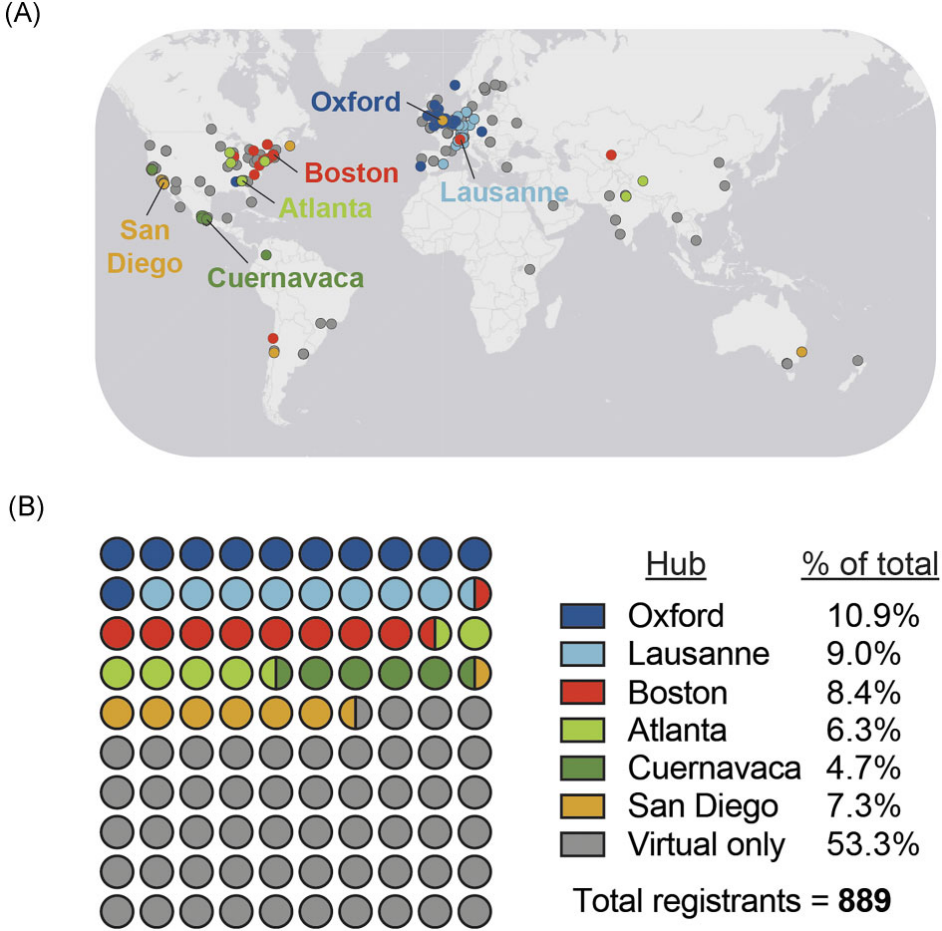
Overview of conference registrants. (a) Map indicating the conference registrants' residence location if it was shared, color-coded by the hub that they attended (see text colors; gray refers to virtual only participation). The six different hubs are indicated on the map by a black line. (b) Distribution of conference registrants across the six different hubs or virtual only participation. The percentage values are indicated on the right and visually represented by color-encoding on the left.

In January 2024, MEEhubs2024, a multi-hub conference on microbial ecology and evolution was held. The conference organizers had originally planned a hub-based conference for 2020 that transitioned to a fully virtual format (MEEvirtual2020) due to concerns about COVID-19. The fully virtual format received significant positive feedback and inspired the conference's continuation in a multi-hub format. MEEhubs2024 was organized across six hubs in five time zones and facilitated local watch parties and individual virtual participation (Fig. 1).

This article takes MEEhubs2024 as a case study and combines participant feedback collected through online surveys with organizer (co-authors of this article) reflections to provide proof of concept for a hybrid multi-hub conference in the microbiology community. We also suggest good practice recommendations to improve multi-hub conferences to assist other conference organizers in accelerating their preparations.

## Materials and methods

### Multi-hub conference logistics

MEEhubs2024 provided a venue for researchers in microbial ecology and evolution to present work to and exchange with a global audience.^1^ The conference took place from 9 to 11 January 2024, following the decision to avoid holidays, other conferences on similar topics, and teaching periods, which expanded participation potential and increased lecture hall availability. Despite aspiring to include as many global regions as possible, logistical considerations restricted this first hub-based edition to Europe and North America, with the goal of future expansion. Accordingly, most in-person registrants were from these regions, although virtual registrants attended from many other locations (Fig. 1).

The core organizing committee comprised five individuals who also represented the European hubs. This group implemented the overall structure of the conference and led the strategic planning. Each North American hub was set up by one or two local hub organizers who worked with the core team. Planning began approximately 18 months prior to the conference. Six months before the conference, a coordinator was hired at an hourly rate to help schedule team meetings, collect notes, organize paperwork, and manage online registration. A few weeks before the conference, each hub recruited volunteers to help with various organizational tasks, with most hubs drawing on local technical staff for audiovisual assistance.

The conference's main sponsor was the Swiss National Centre of Competence in Research Microbiomes, a funding scheme of the Swiss National Science Foundation. The budget covered the costs of the online platform, website, coordinator salary, some travel expenses, and the Lausanne hub. The Microbiology Society offered to associate the meeting with a special collection and sponsored poster prizes for one of the hubs. The UC Irvine Microbiome Center, Don Whitley Scientific, and all hosting universities contributed to local hub costs. This support made it possible to forgo conference registration fees, a decision also motivated by the legal complexities of deploying a shared budget across several countries and institutions.

The six hub locations were selected based on the following criteria: (i) hub organizer availability; (ii) location accessibility, ideally by train; and (iii) geographical locations expected to draw sufficient in-person participation and covering as many time zones as was practical. The choice was also influenced by personal connections from the initiators’ professional networks. In the future, it may be preferable to select time zones and then search for hub organizers longitudinally across the geographical range of each time zone to better distribute hubs. Hub organizers were responsible for locating the venue and organizing lunches, coffee breaks and other local events, such as workshops, conference dinners or screenings of recorded talks. All hubs chose local university campuses as cost-effective venues and provided minimal catering due in part to the lack of registration fees. In addition to the hubs, interested attendees in other locations were encouraged to set up ‘watch parties’ at which several in-person attendees could meet and attend the conference together. The remaining attendees participated individually. To maximize diversity in hub representation, invited and contributing speaker selection considered gender, career stage, and research subdisciplines and methodologies.

The organizers managed registration and communication between hubs using the online platform Oxford Abstracts, ^2^ which provided a website, database, and registration system and facilitated abstract review, scheduling, virtual poster displays, and itinerary generation. This platform provided all attendees with access to Zoom webinars, links to session recordings, an interactive chat platform and the ability to browse the program, abstracts, and posters. The conference was advertised via social media and through the organizers’ professional networks. Upon registration, registrants received information via regular emails and through Oxford Abstracts, as well as two conference guides—general and hub-specific—that included details about venues and local events.

The program was designed so that talks were distributed evenly between the six physical hubs and the virtual speakers, which operated as a seventh hub (Fig. S1, Supplementary Material A). This approach was meant to result in sufficient live talks at each hub to reduce screen fatigue. Invited speakers were given 20 min to present, with 10 min for Q&A, while talks selected from abstracts were 12 min long, with 3 min for Q&A. Speakers at each hub were scheduled between 8 a.m. and 7 p.m. in the time zone of their local hub. All talks were recorded, and sufficient free time was scheduled for attendees to catch up on talks that took place outside of their working hours. At some hubs, these talks were streamed in lecture halls during breaks. In addition to displaying all posters online and facilitating attendee interactions via a messaging feature, physical poster sessions took place at most hubs.

Finally, cross-hub activities were designed to increase engagement across hubs and participation modes. In these hour-long sessions, speakers presented on broader topics and issues in science, such as inclusivity, sustainability, and art in science, and discussed across hubs and the virtual audience, with the help of Mentimeter,^3^ an interactive presentation software.

### Collecting attendee perspectives: pre- and postconference surveys

We conducted a pre- and postconference online survey to collect perspectives from conference attendees. Both surveys were created in Unipark, made available in English, and took around 15 min to complete. Participation was voluntary, and survey participants (hereafter ‘participants’) provided informed consent before starting each survey.^4^

The preconference survey was sent to all conference registrants via email on 3 January 2024 and was advertised on the conference webpage and during the opening session. It remained open until the first conference day. The postconference survey—also advertised on the webpage and during the closing session—was emailed to all registrants on 11 January and closed on 31 January 2024. Participants who selected ‘I did not attend MEEhubs2024’ on the first postconference survey page were excluded from completing the postconference survey.

The preconference survey included questions regarding participants’ perceptions of conferences, their main reasons for attending MEEhubs2024, and their expectations of and skepticism toward the conference and its format. The postconference survey assessed participants’ conference experiences, ideas for improvement and opinions about future conferences in this series. Both surveys collected demographic data and information on participation type (Supplementary Material C). Data analysis—mainly descriptive statistics—was conducted using IBM SPSS Statistics, with Mann–Whitney U tests used to examine differences between in-person and virtual participants due to non-normal variable distributions.

The final samples of the pre- and postconference surveys included 150 participants (16.9% response rate) and 118 participants (13.3% response rate),^5^ respectively. Of the preconference participants, 65.3% identified as female, 29.7% as male, 0.8% as nonbinary/other, and 4.2% did not disclose. In the postconference survey, 55.3% of participants identified as female, 41.3% as male, 1.3% as nonbinary/other, and 2.0% did not disclose. The average age of pre- and postconference participants was 32.1 years (SD = 6.9) and 31.2 years (SD = 7.0), respectively. Further participant demographics are presented in Table 1.

**Table 1.** Survey sample description.

		Preconference survey	Postconference survey
Career stage (multiple choice)	Undergraduate/master student	9	6
	Doctoral/PhD student	52	45
	Postdoc	49	39
	Academic group leader <10 years	27	19
	Academic group leader >10 years	5	5
	Working in industry	2	0
	Clinician	1	1
	Publishing	2	0
	Other	8	5
Scientific field (multiple choice)	Natural/biological sciences	135	116
	Physical sciences/engineering and technology	9	6
	Medical and health sciences	30	22
	Agricultural and veterinary sciences	12	11
	Humanities and arts	1	1
Role of attendance (multiple choice)	Presenter/speaker	67	62
	Attendee only	81	57
	Organizer of a watch-party	4	0
Participation type	In-person attendance	81	86
	Individual virtual attendance	5	1
	Watch-party attendance	63	31

## Results and discussion

### An overall positive conference experience

Before the conference, in open comments, participants mentioned that they wanted to attend MEEhubs2024 due to their interest in the conference's topic and to present, discuss, be inspired by, and learn more about this field, other projects, methods, theories, and news. Participants were also eager to network, meet new and old colleagues and take part in the microbial ecology and evolution community. They were also curious about how the hybrid multi-hub format would work and pointed out that they supported the new format's increased accessibility and reduced carbon footprint. Consistent with findings from other studies (Tao et al. 2021, Moss et al. 2022, Kremser et al. 2023), participants identified the format's environmental friendliness, inclusivity, lower attendance costs, and flexible participation as key benefits. Nevertheless, while hybrid conferences have the potential to increase inclusion, they may still reinforce existing divisions or create new ones if in-person attendance remains limited to a privileged few (Bajpai et al. 2022).

**Figure 2.**
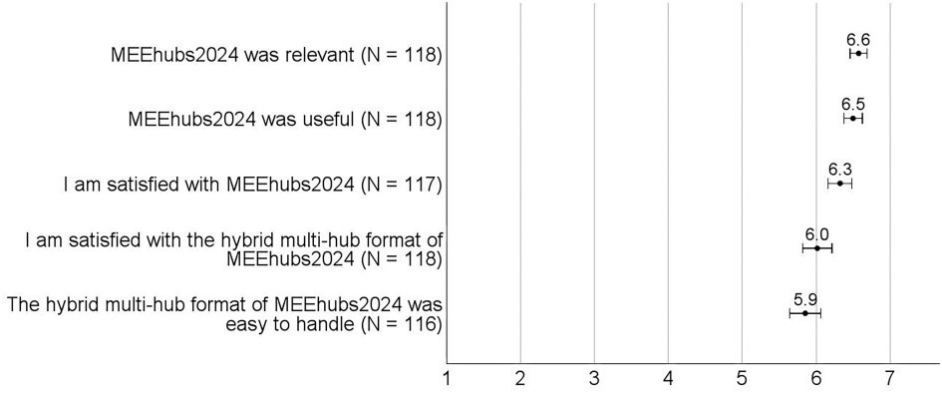
Participants’ overall conference experience. The variables were measured on a Likert scale ranging from 1 (strongly disagree) to 7 (strongly agree). Dots represent means, and error bars represent 95% confidence intervals.

**Figure 3.**
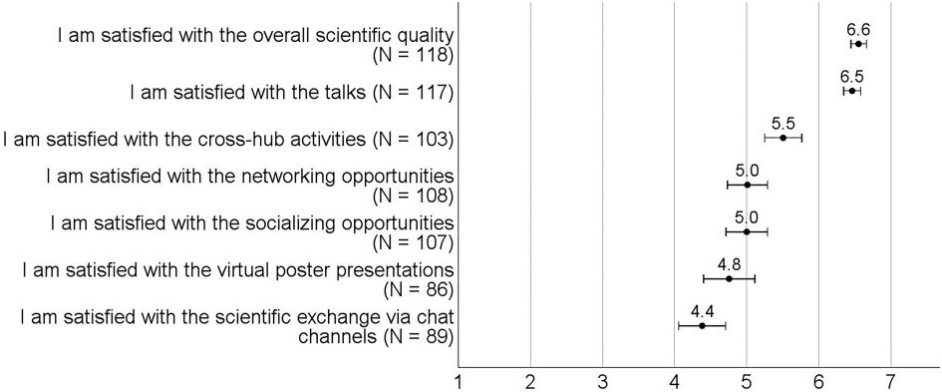
Participants’ satisfaction with different conference elements. The variables were measured on a Likert scale ranging from 1 (strongly disagree) to 7 (strongly agree). Dots represent means, and error bars represent 95% confidence intervals.

After the conference, in open comments, participants described an overall great conference experience, noting that the format worked well and achieved a good balance between global and local community reach. They found the conference relevant, useful and easy to handle, being satisfied with the conference and its format (Fig. 2), particularly its scientific quality and the talks (Fig. 3). Furthermore, participants perceived attending the conference as important for recognition, reputation, their overall career, and in general (Fig. S2, Supplementary Material A). This overall positive experience with MEEhubs2024 was reflected in how many participants answered the question about conference challenges with ‘none’ and commented that the format ran smoothly.

In the postconference survey, in response to a multiple-choice question, most participants (*N* = 111, 74.0%) indicated that they would prefer a hybrid multi-hub format for future conferences on microbial ecology and evolution. The second preferred format was a hybrid conference with one in-person location and a virtual option (*N* = 22, 14.7%), while fully virtual (*N* = 9, 6.0%) and fully in-person (*N* = 8, 5.3%) formats were the least favored. These preferences align with research suggesting that conference attendees favor hybrid formats for future conferences (Puccinelli et al. 2022) and that most attendees would like to attend another multi-hub conference (Kremser et al. 2023). However, hybrid conference attendees may be biased toward this format, which could influence these findings. This potential bias may also explain recent literature indicating a shift back to predominantly in-person conference attendance (Olechnicka et al. 2025).

**Figure 4.**
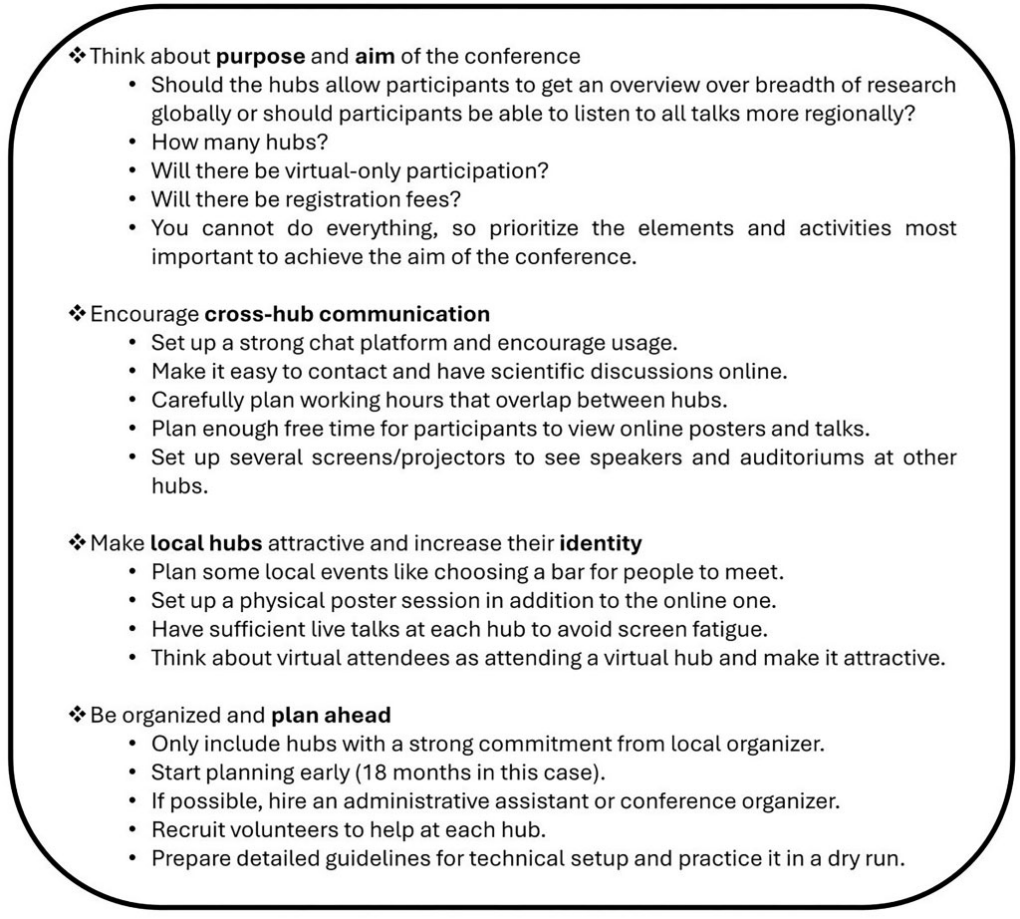
Conference organizers’ key takeaways.

As organizers, we implemented the multi-hub format with the goals of (i) reducing GHG emissions, (ii) increasing inclusivity, (iii) decreasing registration costs, and (iv) reducing organizational costs by using university venues and avoiding long flights for invited speakers. We estimate that these goals were met based on the highly positive feedback from attendees in conversations. We thus consider MEEhubs2024 a success. However, we acknowledge that organizing conferences in this format requires significant time and effort. With hardly any best practices available, we faced various logistical, technical, and structural challenges—some of which we resolved, and others we did not. The recommendations that follow, and the key takeaways presented in Fig. 4 reflect both our successes and the remaining challenges.

### Technical and logistical challenges

Before the conference, survey participants expressed concerns about potential technical issues. Although the conference ran smoothly for the most part, in open comments, postconference participants mentioned several technical challenges, such as low audio and video quality, inability to see other attendees (e.g. those asking questions or raising virtual hands), struggles with the Oxford Abstracts platform, hiccups connecting between hubs, and unstable internet. These issues reflect the challenges reported in other studies on virtual and hybrid conferences (Saatçi et al. 2019, Schwarz et al. 2020, Foramitti et al. 2021). Participants highlighted the need for a better chat platform, which was reflected in the moderate satisfaction reported regarding scientific exchanges via chat (Fig. 3). Satisfaction with the chat was significantly (U = 410.5, Z = −3.1, *P* = .002) lower among in-person participants (M = 4.1, SD = 1.5) than among virtual participants (M = 5.2, SD = 1.3).

Another frequently reported challenge concerned time zone differences. Participants reported that sessions at other hubs were sometimes scheduled at inconvenient times, they struggled to rewatch sessions or catch up on talks at other hubs due to an already full conference schedule and that networking across time zones was perceived as particularly challenging. Time zone issues with virtual and hybrid conferences are well documented in the literature (Foramitti et al. 2021, Puccinelli et al. 2022), which has responded with solutions, including asynchronous conferences, spreading sessions over several days, and making recordings viewable for a longer period (Holman et al. 2021, Moss et al. 2021).

**Figure 5.**
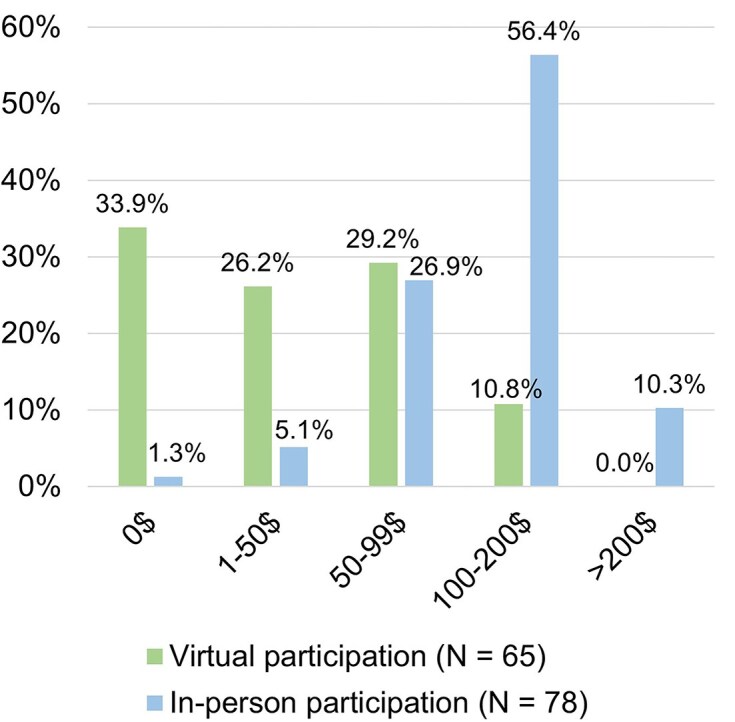
Participants’ indicated values (in US dollars) they would be willing to pay as a registration fee.

Although several participants appreciated free registration, which they perceived as increasing accessibility, some suggested paying a small fee to accommodate more local activities (e.g. meals and social events). Participants were willing to pay more to attend in person, given that most of these events would likely be scheduled in person (Fig. 5). As noted by Molana et al. (2023), free registration also led people to register without actually attending the conference or to switch their registration from in-person to virtual, which increased administrative burdens. Accordingly, we propose two ideas: (i) securing sponsorship, or (ii) requesting a deposit or upfront costs based on attendance type, hub location, and attendee characteristics (e.g. career stage, income) to fund in-person activities. We recommend considering any legal or practical concerns about international fee and deposit collection and distribution as early as possible.

From the organizers’ perspective, switching Zoom hosts between hubs required careful coordination and sufficient time to ensure smooth transitions. Therefore, we suggest that organizers align hub transitions with breaks to allow enough time. To limit technical hiccups, we propose hiring professional or trained technical hosts and using technical tools with high-quality audio and video capabilities. To increase community feeling, we recommend two screens in each conference room: one to display the presentation and another to show the speaker(s). Based on participants’ opinions, we also suggest implementing a chat channel to facilitate attendee interactions. During MEEvirtual2020, attendees enjoyed using Rocket Chat—a platform similar to Slack—to discuss and engage with conference material. However, too many tools and platforms may fragment user engagement, so we advise integrating a minimal number of tools. For more specific good practice recommendations, see Fig. 4 and Supplementary Material B.

### Structural challenges

In-person participants valued face-to-face interactions, informal exchanges and the cozy atmosphere of the hubs. Nevertheless, in open comments, they identified a need to strengthen the local hub identity, citing low attendance at some in-person talks and posters. Some participants reported already knowing many local attendees, which limited new networking opportunities. Overall, the responses revealed a demand for more in-person social events.

Before the conference, participants expressed skepticism about networking opportunities for virtual attendees, which was confirmed in postconference survey comments. Virtual participants reported low engagement with virtual posters and recordings, and some struggled to find time to view virtual posters individually, which informed participants’ moderate satisfaction with virtual poster presentations (Fig. 3). Some virtual participants reported feelings of isolation attributable to the perceived ‘second-class’ experience of virtual attendees compared to in-person attendees, a concern also noted in a previous study (Wenger 2023). The quantitative survey data reflects this perception (Table S1, Supplementary Material A), indicating that virtual participants had fewer formal scientific discussions, less recognition, and fewer breaks from their daily routines than in-person participants (*P*s < .001). To counteract this, previous studies suggested several solutions, such as virtual avatar rooms (e.g. Gather.town) to foster more spontaneous interactions (Wenger 2023), or virtual breakout systems like FluidMeet, which facilitates fluid conversations, group formations, and informal interactions (Hu et al. 2022). Additionally, formats like World Cafés and unconferences (Holman et al. 2021) can encourage more spontaneous discussions and networking opportunities.

Virtual participants perceived networking as the biggest challenge and reported difficulties initiating informal or spontaneous chats, citing that it was impossible to mingle as well as in-person attendees. One participant responded, ‘Walking up to groups to feel the vibes and then contribute to existing conversations with intent listening followed by some banter can only be achieved in an in-person conference’. This perception was reflected in the participants’ moderate satisfaction with networking and socializing opportunities (Fig. 2). Virtual participants also indicated that they had fewer chats and informal exchanges about scientific content and established fewer new professional contacts and social relationships than the in-person participants did (*P*s < .001; Table S1, Supplementary Material A). These issues reflect the challenges reported in studies on virtual conferences (Schwarz et al. 2020, Foramitti et al. 2021).

Before the conference, participants also expressed skepticism about interactions between virtual and in-person attendees due to potential technical malfunctions and gaps between hubs and participation modes. Many participants commented that cross-hub interactions facilitated by tools like Mentimeter worked well; however, networking across hubs and participation modes was difficult due to limited discussion opportunities, low engagement, and insufficient socializing options, especially for virtual attendees, which was reflected in participants' moderate satisfaction with cross-hub activities (Fig. 3).

From the organizers’ perspective, balancing the needs of in-person and virtual attendees was challenging. The organizers endeavored to connect hubs and schedule cross-hub activities, but increasing this feature beyond what was offered would result in higher organizational and logistical complexities. For example, enabling small group chats between in-person and virtual attendees requires dedicated spaces (e.g. small rooms or telephone booths) where local attendees can join Zoom meetings undisturbed. Some participants also expressed a desire for more hubs in other global locations, which would increase time zones and logistical challenges, requiring more coordination between hubs and therefore more organizer administration. We suggest either capping the total number of hubs or rethinking some organizational aspects from scratch.

To strengthen local hub identity, we support participants’ recommendations for an intensive hub program with more in-person sessions and social events. To enhance the sense of virtual community, we agree with participants’ suggestions for more virtual-only socializing sessions that, rather than Zoom's webinar feature, use avatars or virtual reality platforms (see Moss et al. 2021). To increase interactions across hubs and participation modes, we agree with participants’ recommendations that organizers should schedule cross-hub activities and time for unstructured, informal interactions via breakout rooms or one-on-one Zoom meetings when all hubs are online. For more good practice recommendations, see Fig. 4 and Supplementary Material B.

## Conclusion

New conference formats are needed to increase the social and environmental sustainability of carbon-intensive research practices. Hybrid multi-hub conferences, held in several virtually connected in-person venues, offer one way to achieve this by combining the flexibility of virtual participation with face-to-face interactions and socializing (Tao et al. 2021, Moss et al. 2022). This case study of MEEhubs2024—a hybrid multi-hub conference on microbial ecology and evolution—integrated participants’ and organizers’ perspectives, which provided evidence that this novel conference format works and was highly appreciated by participants. Nevertheless, we identified some remaining technical, logistical, and structural challenges. These findings align closely with and expand on the few previous studies of multi-hub conferences (Reshef et al. 2020, Kremser et al. 2023) through an example from the microbial ecology and evolution community. We hope that by providing a template for and good practice recommendations to improve the conference format, this article will help future organizers expedite conference preparations. We also encourage funders and academic institutions to offer more incentives and recognition for organizing conferences in this innovative format.

Several of the difficulties attributed to the hybrid multi-hub conference presented in this article are not unique to this format. Issues such as overloaded conference programs—leading to the feeling of not being able to watch all the talks—technical hiccups, time zone difficulties, fatigue, limited engagement and attendance, and the need for more networking opportunities also apply to in-person and virtual conferences. However, the shortcomings of familiar formats implemented for decades may be overlooked. These concerns prompt reflection on attendees’ assumptions about and expectations of conferences and the collective norms surrounding conference practices. This raises questions, such as: should we reduce the number of talks or shift our expectations that a single-track conference must enable everyone to attend every session? It also challenges us to reconsider what and who we use as a baseline for comparison. For example, is attending virtually better than not participating at all, or is in-person attendance always preferable to virtual participation? These questions highlight a research gap concerning the expectations, norms, and outcomes of academic conferences and emphasize the need for further studies that explore whether attendees and participation modes affect these factors.

Structural challenges revealed that attendees from both participation modes recognized the need to strengthen their sense of community, including informal exchanges and socializing. Although the hybrid format provided greater attendance flexibility, dividing attendees across different hubs and modes at times resulted in low attendance at each, as well as the perception of a lack of networking opportunities within and across hubs and participation modes. The participants reported difficulties in achieving meaningful interactions across hubs and modes, which led them to prefer strengthening connections within their own groups. However, this risks deepening the divide between virtual and in-person attendees and highlights the challenges organizers face in balancing diverse needs and expectations across participation hubs and modes. The findings also highlight a discrepancy between expressed preferences and actual behavior, as we observed limited engagement in the dedicated virtual interaction sessions (i.e. speed-sciencing sessions). Such cognitive dissonance suggests that further research is required to explore why stated desires do not translate into action.

From a practical perspective, these research gaps are associated with a challenge for organizers: for example, and as mentioned above, they may face the choice between hosting a truly global conference with hubs on all continents, but with too much content for attendees to follow, or a more regional conference that allows attendees to view all the talks, but with limited hub locations. Moreover, organizers may need to decide between a hub-focused approach and networking between hubs or between scientific presentations and networking opportunities. While the MEEhubs2024 organizers emphasized the experimental aspect of the conference format, we did not clearly explain all organizational decisions. Transparency may help attendees better appreciate that not all aspects of a conference can be optimized simultaneously.

This study was limited in the sense that the results are specific to one multi-hub conference on microbial ecology and evolution held at six locations across Europe and North America. Thus, several survey questions were specific to MEEhubs2024’s setup and structure. Moreover, our recommendations are most applicable to conferences of a similar size. Scaling the format up or down may require rethinking aspects such as the number and distribution of hubs. While certain efforts, like setting up technical equipment and having available infrastructure, remain consistent regardless of conference size, the balance between organizational efforts and the potential attendee density near the hubs must be carefully evaluated to ensure the feasibility of the multi-hub format. There were also organizational and logistical differences between the six hubs for which we did not account, and the conference took place directly after holidays in the various hub locations, which might have affected some of the responses and conference organization (i.e. communication and preparation prior to the conference). Finally, we did not assess the participants’ prior experience with virtual and hybrid conference formats. We suggest that future research considers how prior experience with the format might affect participants’ experiences.

We hope that the multi-hub format will continue to be replicated, evaluated and scaled up with professional conference companies serving as a new standard conference model. We call on the community and conference organizers to test and evaluate innovative tools and formats, such as the Conect application developed by Secret et al. (2021), to better connect in-person and virtual attendees. It is essential for the microbial ecology and evolution community to leverage the momentum and knowledge gained from MEEhubs2024 to organize more multi-hub conferences, thereby acquiring organizational skills and reinforcing new community norms that support a shift toward more sustainable conference practices.

## Supplementary Material

fnaf022_Supplemental_Files

## Footnotes

1
https://www.meehubs.org or https://virtual.oxfordabstracts.com/event/4373/information

2
www.oxfordabstracts.com

3
www.mentimeter.com

4 The study design was reviewed and approved by the ETH Zurich Ethics Commission (EK-2023-N-324).

5 Survey response rates were calculated based on the total number of registrants (N = 889); however, we do not know how many actually attended one or more conference sessions.
