# Supplementary material for: MEEhubs2024: A hub-based conference on microbial ecology and evolution fostering sustainability
Source: FEMS Microbiol Lett. 2025 Feb 6;372:fnaf022. doi: 10.1093/femsle/fnaf022 (PMC11879406; doi:10.1093/femsle/fnaf022)
Supplement: fnaf022_Supplemental_Files [file fnaf022_supplemental_files.zip › Supplementary_Material_clean.pdf]

SUPPLEMENTARY MATERIAL

MEEhubs2024: A hub-based conference on microbial ecology and evolution fostering sustainability

Ariane Wenger *, Erik Bakkeren, Elisa Granato, Robin Tecon, Sara Mitri, Wolfram Möbius

FEMS Microbiology Letters

*Corresponding author
Ariane Wenger
TdLab
Universitätsstrasse 16
8092 Zurich, Switzerland
ariane.wenger@usys.ethz.ch

Supplementary Material A: Supplementary Tables and Figures

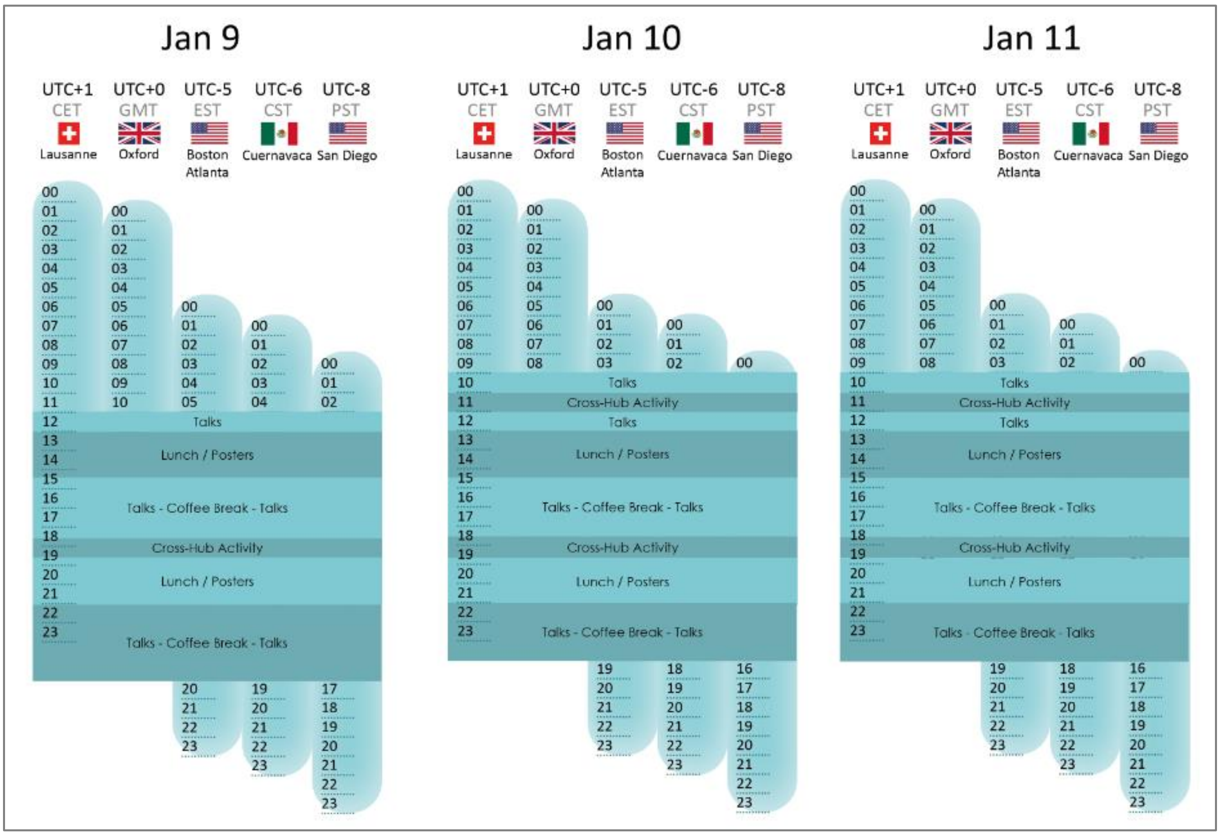

Figure S1. Overview of the MEEhubs2024 conference schedule.

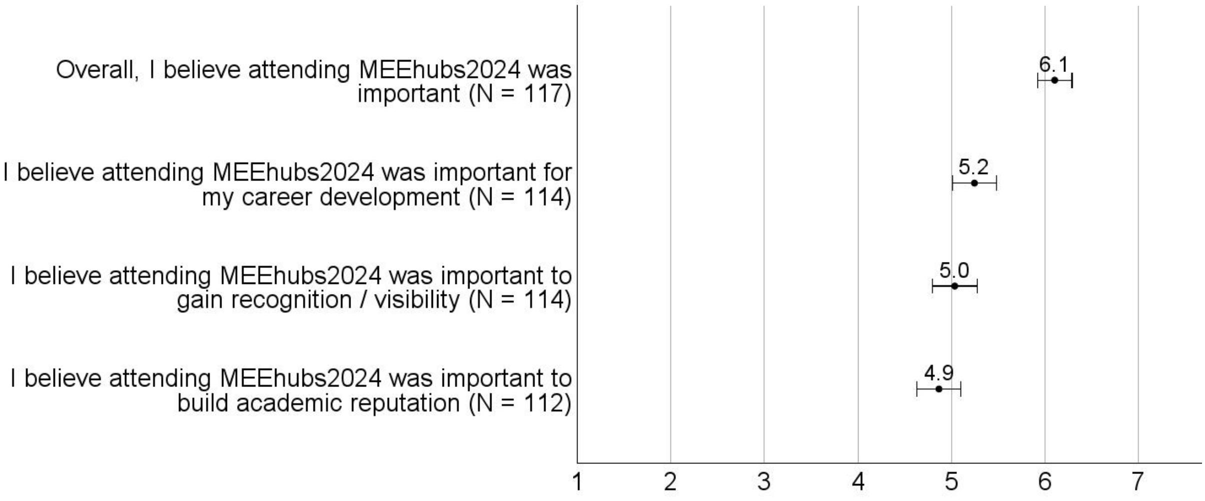

Figure S2. Perceived importance of attending MEEhubs2024.
The variables were measured on a Likert scale ranging from 1 (strongly disagree) to 7 (strongly agree). Dots represent means, and error bars represent 95% confidence intervals.

Table S1. Differences in benefits gained between virtual and in-person attendees.

	**Mann-Whitney-U test**			**Mean**	
	**U**	**Z**	**p**	**Virtual**	**In-person**
I established (a) new professional contact(s).	554.5	-4.3	< 0.001	3.2	5.0
I established (a) new social relationship.	544.5	-4.4	< 0.001	2.8	4.8
I had formal discussion of scientific content.	422.5	-5.3	< 0.001	3.4	5.5
I had chats and informal exchanges on scientific content.	253.5	-6.5	< 0.001	3.4	6.0
I gained recognition / visibility.	770.0	-3.3	< 0.001	3.5	4.6
I got a break from my daily routine(s).	700.5	-3.7	< 0.001	5.2	6.1

Supplementary Material B: Good practice recommendations

The following list of good practice recommendations for organizing hybrid multi-hub conferences stems from survey participants' suggestions and our - MEEhubs2024 organizers' - reflections.

Solving logistical challenges

- Do not schedule back-to-back sessions hosted by different hubs, but allow time for transition between hubs, e.g., by aligning hub transitions with breaks.
- Do not schedule too many talks a day and shorten the conference day to allow for in-between time (e.g., to catch up with sessions from other hubs).
- Stream the sessions from other hubs outside of a hub's time zone during long breaks.
- Plan as many slots for all hubs together as possible.
- Plan the keynote talk(s) at a time when the overlap of hubs is maximized. Carefully decide on the purpose of keynote talks and whether most/all hubs should be able to participate.
- Do not use pre-recorded talks unless no other option exists. For accessibility, real-time transcription can be used.
- Consider incentives that reduce the number of no-shows or last-minute changes of registration to help planning and reduce administrative burden.

Solving technical challenges

- Communicate with the local hub organizers and technical hosts early and frequently.
- Prior to the conference, schedule testing sessions with the speakers to check that everything works technically and test all microphones and cameras used regarding their compatibility and quality on Zoom. Good audio and video quality are paramount!
- Have professionals or trained students as technical hosts in each conference room and assign them specific roles.
- During the conference, use an instant messaging platform to communicate among organizers and technical hosts in cases of on-spot technical issues.
- Provide a short guide for on-spot technical troubleshooting in each conference room.
- In the conference rooms, use the zoom function of the cameras (where available) to zoom in on the speakers and people talking/asking questions.
- Use two screens in each conference room: one that shows the speaker(s), and one that shows the presentation.
- Use a conference platform that provides extensive search options.
- Provide a Rocket Chat or Slack workspace in addition to the conference platform to allow attendees to exchange thoughts during the conference.
- Prioritize web browser software and choose platforms/tools that are both free for attendees and compatible with various operating systems, while also considering data protection regulations to safeguard attendees' privacy.
- Inform attendees in advance about any software requirements, ensuring that they are aware of how these tools can be used.

Solving structural challenges

Local hub identity

- Schedule many in-person activities, such as introduction rounds, food and drinks, coffee breaks, evening receptions, and social events.
- Schedule many live sessions, local talks, and workshops at each hub throughout the day to have an intensive hub program.
- Plan an in-person poster session to enhance networking opportunities.

Virtual community feeling

- Schedule and organize online-only events, sessions, and social activities for the virtual attendees. Encourage in-person attendees to join these virtually.
- Provide interactive activities via more versatile chat platforms - e.g., those using avatars - or a virtual reality platform.
- Specifically schedule a session (allocating time and space) to watch and comment on the virtual posters. Poster presenters can explain their posters in short pitches to increase engagement live or in prerecorded snippets.
- Implement discussion sessions of the most "interesting" posters or implement a poster award to increase engagement with the virtual posters.
- Do not use the webinar function of Zoom or choose settings so that attendees can see each other, raise a virtual hand, and use emojis.
- Regularly advertise and encourage attendees to comment on the recordings and posters.
- Introduce the norm that attendees mention their name and institution before asking questions.

Interactions across participation modes/hubs

- Increase the time where all hubs are online to the fullest and use it for cross-hub activities, such as topic-specific panels and discussions. Use a platform like Mentimeter to maximize engagement.
- Schedule sessions for smaller groups, use breakout rooms, or slots for one-on-one Zoom meetings across hubs/participation mode.
- Schedule more unstructured sessions to interact informally and plan frequent breaks to allow time for informal discussions with speakers.
- On the conference webpage, include an easily searchable list of attendees (e.g., by adding individual keywords or hashtags).
- Show the chat with the questions on one screen at each hub.
- Constantly stream the view of the other hub(s) on one screen.
- Take photos from the different hubs and share them across all attendees.
- Create hashtags for the conference to use on social media.

Supplementary Material C: Surveys

C1 - Pre conference survey

Welcome and thank you for your participation!

This survey is conducted to assess the perceived situation and opinions of the MEEhubs24 attendees. It is also part of a study conducted within a dissertation on academic air travel reduction and virtual communication. Filling out the survey will help capture attendees' perspectives and expectations of the MEEhubs24 conference.

The survey takes about 10-15 minutes to complete. Participation is voluntary, and you can withdraw from the survey at any time by closing your browser. In this case, your entered data will not be analyzed. All information you provide will be stored and evaluated in an anonymized form. At the end of the survey you can indicate whether you would like your results to be used only as feedback to conference organisers or also for research purposes.

Please note that your participation in the MEEhubs24 conference does not imply an expectation or a demand to respond to this survey.

For further inquiries about the survey, please contact Ariane Wenger (TdLab, ETH Zurich) at ariane.wenger@usys.ethz.ch.

This survey was reviewed and approved by the ETH Zurich Ethics Commission (EK 2023-N-324).

☐ I voluntarily participate in this survey and agree to the processing of my data in accordance with the above-mentioned information.

I don't want to participate *START THE SURVEY*

Please note three words that spontaneously come to your mind when thinking about what is important at academic conferences: ____

How important do you perceive conferences to be for you?

	1 Strongly disagree	2	3	4	5	6	7 Strongly agree	Prefer not to say
Overall, I believe conference attendance is important. *(pre_v1)*	☐	☐	☐	☐	☐	☐	☐	☐
I believe conference attendance is important for my career development. *(pre_v2)*	☐	☐	☐	☐	☐	☐	☐	☐
I believe conference attendance is important to build academic reputation. *(pre_v3)*	☐	☐	☐	☐	☐	☐	☐	☐
I believe conference attendance is important to gain recognition / visibility. *(pre_v4)*	☐	☐	☐	☐	☐	☐	☐	☐

If you would like to elaborate on the perceived importance of conferences, you may do so here (optional): ____

What is your general perception of the following conference formats?

	1 Very negative	2	3	4	5	6	7 Very positive	Don't know / Prefer not to say
In-person conferences. *(pre_v5)*	☐	☐	☐	☐	☐	☐	☐	☐
Hybrid conferences held at one in-person location + virtual participation. *(pre_v6)*	☐	☐	☐	☐	☐	☐	☐	☐
Hybrid conferences held at multiple in-person hubs + virtual participation. *(pre_v7)*	☐	☐	☐	☐	☐	☐	☐	☐
Virtual conferences. *(pre_v8)*	☐	☐	☐	☐	☐	☐	☐	☐

In general, how optimistic are you that virtual conferences will improve in the near future (i.e., within the next five years) in terms of technological tools used and their organization / structure? *(pre_v9)*

☐ Not at all optimistic
☐ Somewhat not optimistic
☐ Neither
☐ Somewhat optimistic
☐ Optimistic
☐ Very optimistic
☐ Prefer not to say

If you would like to explain your opinion on different conference formats, you may do so here (optional): ____

What is the main reason why you are attending MEEhubs24? _______________________

How did you hear about MEEhubs24?

☐ Social media *(pre_v10)*
☐ Colleagues *(pre_v11)*
☐ MEEhubs website *(pre_v12)*
☐ Mailing list *(pre_v13)*
☐ Other (please specify) *(pre_v14)*
☐ Prefer not to say *(pre_v15)*

In which role are you attending MEEhubs24?

☐ Presenter / Speaker *(pre_v16)*
☐ Attendee only *(pre_v17)*
☐ Organizing committee (main organizer / organizer of a hub) *(pre_v18)*
☐ Organizer of a watch-party *(pre_v19)*
☐ Prefer not to say *(pre_v20)*

How do you plan on participating in MEEhubs24? *(pre_v21)*

☐ In-person attendance at one of the hubs
☐ Watch-party attendance
☐ Individual virtual attendance
☐ Prefer not to say

If you would like to explain the reasons for the type of participation in MEEhubs24, you may do so here (optional): ____

When thinking about the format of MEEhubs24 and the possibilities this conference provides, what are you looking forward to and what are you sceptical about (optional)? ______________________________

How important do you perceive MEEhubs24 to be for you?

	1<br>Strongly disagree	2	3	4	5	6	7<br>Strongly agree	Prefer not to say
Overall, I believe attending MEEhubs24 is important. *(pre_v22)*	☐	☐	☐	☐	☐	☐	☐	☐
I believe attending MEEhubs24 is important for my career development. *(pre_v23)*	☐	☐	☐	☐	☐	☐	☐	☐
I believe attending MEEhubs24 is important to build academic reputation. *(pre_v24)*	☐	☐	☐	☐	☐	☐	☐	☐
I believe attending MEEhubs24 is important to gain recognition / visibility. *(pre_v25)*	☐	☐	☐	☐	☐	☐	☐	☐

If you would like to elaborate on the perceived importance of MEEhubs24, you may do so here (optional): ____

Which of the following do you expect to gain from attending MEEhubs24?

	1 Strongly disagree	2	3	4	5	6	7 Strongly agree	Prefer not to say
Re-establishing (an) old professional contact(s). *(pre_v26)*	☐	☐	☐	☐	☐	☐	☐	☐
Establishing (a) new professional contact(s). *(pre_v27)*	☐	☐	☐	☐	☐	☐	☐	☐
Establishing (a) new social relationship(s). *(pre_v28)*	☐	☐	☐	☐	☐	☐	☐	☐
Initiating / establishing a research collaboration with someone. *(pre_v29)*	☐	☐	☐	☐	☐	☐	☐	☐
Opportunities to connect with people from the same hub. *(pre_v30)*	☐	☐	☐	☐	☐	☐	☐	☐
Opportunities to connect with people from other hubs or who participate fully virtual. *(pre_v31)*	☐	☐	☐	☐	☐	☐	☐	☐
Formal discussions of scientific content. *(pre_v32)*	☐	☐	☐	☐	☐	☐	☐	☐
Chats and informal exchanges on scientific content. *(pre_v33)*	☐	☐	☐	☐	☐	☐	☐	☐

Which of the following do you expect to gain from attending MEEhubs24?

	1 Strongly disagree	2	3	4	5	6	7 Strongly agree	Prefer not to say
Acquiring new scientific knowledge. *(pre_v34)*	☐	☐	☐	☐	☐	☐	☐	☐
Receiving job-related information (e.g., vacancies or job changes by others). *(pre_v35)*	☐	☐	☐	☐	☐	☐	☐	☐
Receiving information on relevant events (e.g., workshops, further education, conferences). *(pre_v36)*	☐	☐	☐	☐	☐	☐	☐	☐
Receiving tips on funding opportunities (e.g., scholarships, funding organizations). *(pre_v37)*	☐	☐	☐	☐	☐	☐	☐	☐

Which of the following do you expect to gain from attending MEEhubs24?

	1 Strongly disagree	2	3	4	5	6	7 Strongly agree	Prefer not to say
Ideas for new research / a new project. *(pre_v38)*	☐	☐	☐	☐	☐	☐	☐	☐
Learning about a new theory, approach, or method that I can use in my work. *(pre_v39)*	☐	☐	☐	☐	☐	☐	☐	☐
A new personal skill (e.g., presentation or communication skill). *(pre_v40)*	☐	☐	☐	☐	☐	☐	☐	☐
Receiving helpful feedback that can improve my work / research. *(pre_v41)*	☐	☐	☐	☐	☐	☐	☐	☐
Learning about other projects. *(pre_v42)*	☐	☐	☐	☐	☐	☐	☐	☐
Getting an overview of the scientific field of microbial ecology and evolution. *(pre_v43)*	☐	☐	☐	☐	☐	☐	☐	☐
Identifying current scientific trends. *(pre_v44)*	☐	☐	☐	☐	☐	☐	☐	☐

Which of the following do you expect to gain from attending MEEhubs24?

	1 Strongly disagree	2	3	4	5	6	7 Strongly agree	Prefer not to say
Recognition / visibility. *(pre_v45)*	☐	☐	☐	☐	☐	☐	☐	☐
Reputation. *(pre_v46)*	☐	☐	☐	☐	☐	☐	☐	☐
Advancing career / CV. *(pre_v47)*	☐	☐	☐	☐	☐	☐	☐	☐
Receiving a job offer. *(pre_v48)*	☐	☐	☐	☐	☐	☐	☐	☐
Contributing to the advancement of the scientific field of microbial ecology and evolution. *(pre_v49)*	☐	☐	☐	☐	☐	☐	☐	☐

Which of the following do you expect to gain from attending MEEhubs24?

	1 Strongly disagree	2	3	4	5	6	7 Strongly agree	Prefer not to say
An idea, a contact, or information that leads to a new publication. *(pre_v50)*	☐	☐	☐	☐	☐	☐	☐	☐
An idea, a contact, or information that leads to a new grant proposal. *(pre_v51)*	☐	☐	☐	☐	☐	☐	☐	☐
The chance to be a reviewer for a journal. *(pre_v52)*	☐	☐	☐	☐	☐	☐	☐	☐
The chance to work as a guest researcher at another institution. *(pre_v53)*	☐	☐	☐	☐	☐	☐	☐	☐
The chance to give a presentation at another institution. *(pre_v54)*	☐	☐	☐	☐	☐	☐	☐	☐

Which of the following do you expect to gain from attending MEEhubs24?

	1 Strongly disagree	2	3	4	5	6	7 Strongly agree	Prefer not to say
Chance to visit the conference site / doing sightseeing. *(pre_v55)*	☐	☐	☐	☐	☐	☐	☐	☐
Getting a break from my daily routine(s). *(pre_v56)*	☐	☐	☐	☐	☐	☐	☐	☐

If you would like to elaborate on the expected gains from MEEhubs24, you may do so here (optional):

What is your current career stage?
☐ Undergraduate / master's student
☐ Doctoral / PhD student
☐ Postdoc
☐ Academic group leader < 3 years
☐ Academic group leader 3 – 10 years
☐ Academic group leader > 10 years
☐ Working in industry
☐ Clinician
☐ Publishing
☐ Other (please specify)
☐ Prefer not to say

In which scientific field do you work?
☐ Natural sciences *(pre_v57)*
☐ Engineering and technology *(pre_v58)*
☐ Medical and health sciences *(pre_v59)*
☐ Agricultural and veterinary sciences *(pre_v60)*
☐ Humanities and arts *(pre_v61)*
☐ Social sciences *(pre_v62)*
☐ Other (please specify) *(pre_v63)*
☐ Prefer not to say *(pre_v64)*

Which approach / methodology is relevant for your work?
☐ Bioinformatics *(pre_v65)*
☐ Clinical study *(pre_v66)*
☐ Experimental – in vitro *(pre_v67)*
☐ Experimental – in vivo *(pre_v68)*
☐ Field work *(pre_v69)*
☐ Theory / modelling *(pre_v70)*
☐ Other (please specify) *(pre_v71)*
☐ Prefer not to say *(pre_v72)*

In which country are you currently employed?
☐ ______________
☐ Prefer not to say

What is your nationality?
☐ ______________
☐ Prefer not to say

How old are you?
☐ ______________
☐ Prefer not to say

With which gender do you identify?
☐ Female
☐ Male
☐ Non-binary / other
☐ Prefer not to say

If you would like to add any final comments on the topics covered by this survey or the survey itself, you may do so here (optional): ____

Please indicate below, if you agree that your survey entries are used for research purposes (the dissertation on academic air travel reduction and virtual communication):
☐ Yes, my survey entries may be used for research purposes and as feedback to conference organisers.
☐ No, I wish that my survey entries are solely used for feedback to conference organisers and not for research purposes.

C2 - Post conference survey

Welcome and thank you for your participation!

This survey is conducted to assess the perceived situation and opinions of MEEhubs24 attendees after the conference. It is also part of a study conducted within a dissertation on academic air travel reduction and virtual communication. Filling out the survey will help capture attendees' experiences with and evaluation of MEEhubs24.

The survey takes about 15 minutes to complete. Participation is voluntary, and you can withdraw from the survey at any time by closing your browser. In this case, your entered data will not be analyzed. All information you provide will be stored and evaluated in an anonymized form. At the end of the survey you can indicate whether you would like your results to be used only as feedback to conference organisers or also for research purposes.

Please note that your participation in MEEhubs24 does not imply an expectation or a demand to respond to this survey.

For further inquiries about the survey, please contact Ariane Wenger (TdLab, ETH Zurich) at ariane.wenger@usys.ethz.ch.

This survey was reviewed and approved by the ETH Zurich Ethics Commission (EK 2023-N-324).

☐ I voluntarily participate in this survey and agree to the processing of my data in accordance with the above-mentioned information.

I don't want to participate *START THE SURVEY*

In which role are you attending MEEhubs24?
☐ Presenter / Speaker *(post_v1)*
☐ Attendee only *(post_v2)*
☐ Organizing committee (main organizer / organizer of a hub) *(post_v3)*
☐ Organizer of a watch-party *(post_v4)*
☐ Prefer not to say *(post_v5)*
☐ I did not attend MEEhubs24 *(filtered out)*

How did you participate in MEEhubs24? *(post_v6)*
☐ In-person attendance at one of the hubs
☐ Watch-party attendance
☐ Individual virtual attendance
☐ Prefer not to say

At which hub did you attend MEEhubs24? *(post_v7)*
☐ Lausanne, Switzerland
☐ Oxford, United Kingdom
☐ Boston, USA Northeast
☐ Atlanta, USA Southeat
☐ Cuernavaca, Mexico
☐ San Diego, USA West
☐ Prefer not to say

What were the biggest challenges for participating in the hybrid, multi-hub format of MEEhubs24? ___

How would you rate the overall experience of MEEhubs24?

	1 Strongly disagree	2	3	4	5	6	7 Strongly agree	Do not know / Prefer not to say
I am satisfied with MEEhubs24. *(post_v8)*	☐	☐	☐	☐	☐	☐	☐	☐
MEEhubs24 was useful. *(post_v9)*	☐	☐	☐	☐	☐	☐	☐	☐
MEEhubs24 was relevant. *(post_v10)*	☐	☐	☐	☐	☐	☐	☐	☐
MEEhubs24 was of good scientific quality. *(post_v11)*	☐	☐	☐	☐	☐	☐	☐	☐
I am satisfied with the hybrid, multi-hub format of MEEhubs24. *(post_v12)*	☐	☐	☐	☐	☐	☐	☐	☐
The hybrid, multi-hub format of MEEhubs24 was easy to handle. *(post_v13)*	☐	☐	☐	☐	☐	☐	☐	☐
Compared to a fully in-person conference, MEEhubs24 was more environmentally friendly. *(post_v14)*	☐	☐	☐	☐	☐	☐	☐	☐
Compared to a fully in-person conference, MEEhubs24 was more inclusive. *(post_v15)*	☐	☐	☐	☐	☐	☐	☐	☐
Compared to a fully virtual conference, MEEhubs24 was more environmentally friendly. *(post_v16)*	☐	☐	☐	☐	☐	☐	☐	☐
Compared to a fully virtual conference, MEEhubs24 was more inclusive. *(post_v17)*	☐	☐	☐	☐	☐	☐	☐	☐

How would you rate the following aspects of MEEhubs24?
I am satisfied with…

	1 Strongly disagree	2	3	4	5	6	7 Strongly agree	Do not know / Prefer not to say
… talks. *(post_v18)*	☐	☐	☐	☐	☐	☐	☐	☐
…cross-hub activities. *(post_v19)*	☐	☐	☐	☐	☐	☐	☐	☐
…local poster presentations. *(post_v20)*	☐	☐	☐	☐	☐	☐	☐	☐
…virtual poster presentations. *(post_v21)*	☐	☐	☐	☐	☐	☐	☐	☐
…networking opportunities. *(post_v22)*	☐	☐	☐	☐	☐	☐	☐	☐
...socializing opportunities. *(post_v23)*	☐	☐	☐	☐	☐	☐	☐	☐
…scientific exchange via chat channels. *(post_v24)*	☐	☐	☐	☐	☐	☐	☐	☐
…communication prior to the conference. *(post_v25)*	☐	☐	☐	☐	☐	☐	☐	☐
…overall scientific quality. *(post_v26)*	☐	☐	☐	☐	☐	☐	☐	☐
…support by the organizing committee. *(post_v27)*	☐	☐	☐	☐	☐	☐	☐	☐

How did you experience the balance of cross-hub activities and local hub activities? *(post_v28)*
☐ There were too many cross-hub activities in comparison to local hub activities.
☐ There were somewhat too many cross-hub activities in comparison to local hub activities.
☐ There was the right balance between cross-hub activities and local hub activities.
☐ There were somewhat too many local hub activities in comparison to cross-hub activities.
☐ There were too many local hub activities in comparison to cross-hub activities.
☐ Prefer not to say

If you would like to elaborate on your experience of attending MEEhubs24, you may do so here (e.g., which aspects were unsatisfactory / satisfactory and why) (optional): ____________________________

If you remember what you were skeptical about in the first survey conducted before the conference, has your perception changed on this (e.g., regarding the conference format or possibilities it provided) (optional)? ____

How important do you think MEEhubs24 was for you?

	1 Strongly disagree	2	3	4	5	6	7 Strongly agree	Prefer not to say
Overall, I believe attending MEEhubs24 was important. *(post_v29)*	☐	☐	☐	☐	☐	☐	☐	☐
I believe attending MEEhubs24 was important for my career development. *(post_v30)*	☐	☐	☐	☐	☐	☐	☐	☐
I believe attending MEEhubs24 was important to build academic reputation. *(post_v31)*	☐	☐	☐	☐	☐	☐	☐	☐
I believe attending MEEhubs24 was important to gain recognition / visibility. *(post_v32)*	☐	☐	☐	☐	☐	☐	☐	☐

If you would like to elaborate on the perceived importance of MEEhubs24, you may do so here (optional): ____

Which of the following benefits did you gain from attending MEEhubs24?

	1 Strongly disagree	2	3	4	5	6	7 Strongly agree	Prefer not to say
I re-established (an) old professional contact(s). *(post_v33)*	☐	☐	☐	☐	☐	☐	☐	☐
I established (a) new professional contact(s). *(post_v34)*	☐	☐	☐	☐	☐	☐	☐	☐
I established (a) new social relationship(s). *(post_v35)*	☐	☐	☐	☐	☐	☐	☐	☐
I initiated / established a research collaboration with someone. *(post_v36)*	☐	☐	☐	☐	☐	☐	☐	☐
I was able to connect with people from the same hub. *(post_v37)*	☐	☐	☐	☐	☐	☐	☐	☐
I was able to connect with people from other hubs or who participated fully virtual. *(post_v38)*	☐	☐	☐	☐	☐	☐	☐	☐
I had formal discussions of scientific content. *(post_v39)*	☐	☐	☐	☐	☐	☐	☐	☐
I had chats and informal exchanges on scientific content. *(post_v40)*	☐	☐	☐	☐	☐	☐	☐	☐

Which of the following benefits did you gain from attending MEEhubs24?

	1 Strongly disagree	2	3	4	5	6	7 Strongly agree	Prefer not to say
I acquired new scientific knowledge. *(post_v41)*	☐	☐	☐	☐	☐	☐	☐	☐
I received job-related information (e.g., vacancies or job changes by others). *(post_v42)*	☐	☐	☐	☐	☐	☐	☐	☐
I received information on relevant events (e.g., workshops, further education, conferences). *(post_v43)*	☐	☐	☐	☐	☐	☐	☐	☐
I received tips on funding opportunities (e.g., scholarships, funding organizations). *(post_v44)*	☐	☐	☐	☐	☐	☐	☐	☐

Which of the following benefits did you gain from attending MEEhubs24?

	1 Strongly disagree	2	3	4	5	6	7 Strongly agree	Prefer not to say
I gained ideas for new research / a new project. *(post_v45)*	☐	☐	☐	☐	☐	☐	☐	☐
I learnt about a new theory, approach, or method that I can use in my work. *(post_v46)*	☐	☐	☐	☐	☐	☐	☐	☐
I gained a new personal skill (e.g., presentation or communication skill). *(post_v47)*	☐	☐	☐	☐	☐	☐	☐	☐
I received helpful feedback that can improve my work / research. *(post_v48)*	☐	☐	☐	☐	☐	☐	☐	☐
I learnt about other projects. *(post_v49)*	☐	☐	☐	☐	☐	☐	☐	☐
I got an overview of the scientific field of microbial ecology and evolution. *(post_v50)*	☐	☐	☐	☐	☐	☐	☐	☐
I identified current scientific trends. *(post_v51)*	☐	☐	☐	☐	☐	☐	☐	☐

Which of the following benefits did you gain from attending MEEhubs24?

	1 Strongly disagree	2	3	4	5	6	7 Strongly agree	Prefer not to say
I gained recognition / visibility. *(post_v52)*	☐	☐	☐	☐	☐	☐	☐	☐
I gained reputation. *(post_v53)*	☐	☐	☐	☐	☐	☐	☐	☐
I advanced my career / CV. *(post_v54)*	☐	☐	☐	☐	☐	☐	☐	☐
I received a job offer. *(post_v55)*	☐	☐	☐	☐	☐	☐	☐	☐
I contributed to the advancement of the scientific field of microbial ecology and evolution. *(post_v56)*	☐	☐	☐	☐	☐	☐	☐	☐

Which of the following benefits did you gain from attending MEEhubs24?

	1 Strongly disagree	2	3	4	5	6	7 Strongly agree	Prefer not to say
I gained an idea, a contact, or information that leads to a new publication. *(post_v57)*	☐	☐	☐	☐	☐	☐	☐	☐
I gained an idea, a contact, or information that leads to a new grant proposal. *(post_v58)*	☐	☐	☐	☐	☐	☐	☐	☐
I gained the chance to be a reviewer for a journal. *(post_v59)*	☐	☐	☐	☐	☐	☐	☐	☐
I gained the chance to work as a guest researcher at another institution. *(post_v60)*	☐	☐	☐	☐	☐	☐	☐	☐
I gained the chance to give a presentation at another institution. *(post_v61)*	☐	☐	☐	☐	☐	☐	☐	☐

Which of the following benefits did you gain from attending MEEhubs24?

	1 Strongly disagree	2	3	4	5	6	7 Strongly agree	Prefer not to say
I visited the conference site / did sightseeing. *(post_v62)*	☐	☐	☐	☐	☐	☐	☐	☐
I got a break from my daily routine(s). *(post_v63)*	☐	☐	☐	☐	☐	☐	☐	☐

If you would like to elaborate on the benefits gained from attending MEEhubs24, you may do so here (optional): ____

Do you have any suggestions or ideas on how to improve MEEhubs conferences (e.g., networking chances, knowledge exchange, etc.) (optional)? ____

What do you think it would have been worth paying to participate in MEEhubs24?
Virtual registration: _____________ *(post_v64)*
Hub registration: _______________ *(post_v65)*

How would you like future MEE conferences to be held?
☐ Fully in-person *(post_v66)*
☐ Fully virtual *(post_v67)*
☐ Hybrid: One in-person conference location + virtual participation *(post_v68)*
☐ Hybrid: Multiple in-person conference hubs across the world + virtual participation *(post_v69)*
☐ Prefer not to say *(post_v70)*

How would you like the balance of cross-hub activities and local hub activities to be at a future MEEhubs conference? *(post_v71)*
☐ More cross-hub activities than local hub activities.
☐ Somewhat more cross-hub activities than local hub activities.
☐ Same amount of cross-hub activities and local hub activities.
☐ Somewhat more local hub activities than cross-hub activities.
☐ More local hub activities than cross-hub activities.
☐ Prefer not to say

Would you like to see another MEE conference held in a hybrid, multi-hub or a similar format organized in 2026? *(post_v72)*
☐ Yes
☐ No
☐ Prefer not to say

What is your current career stage?
☐ Undergraduate / master's student
☐ Doctoral / PhD student
☐ Postdoc
☐ Academic group leader < 3 years
☐ Academic group leader 3 – 10 years
☐ Academic group leader > 10 years
☐ Working in industry
☐ Clinician
☐ Publishing
☐ Other (please specify)
☐ Prefer not to say

In which scientific field do you work?
☐ Biological science *(post_v73)*
☐ Physical science (including engineering and mathematics *(post_v74)*
☐ Medical and health sciences *(post_v75)*
☐ Agricultural and veterinary sciences *(post_v76)*
☐ Humanities and arts *(post_v77)*
☐ Social sciences *(post_v78)*
☐ Other (please specify) *(post_v79)*
☐ Prefer not to say *(post_v80)*

Which approach / methodology is relevant for your work?
☐ Bioinformatics *(post_v81)*
☐ Clinical study *(post_v82)*
☐ Experimental – in vitro *(post_v83)*
☐ Experimental – in vivo *(post_v84)*
☐ Field work *(post_v85)*
☐ Theory / modelling *(post_v86)*
☐ Other (please specify) *(post_v87)*
☐ Prefer not to say *(post_v88)*

In which country are you currently employed?
☐ _____________
☐ Prefer not to say

What is your nationality?
☐ _____________
☐ Prefer not to say

How old are you?
☐ _____________
☐ Prefer not to say

With which gender do you identify?
☐ Female
☐ Male
☐ Non-binary / other
☐ Prefer not to say

If you would like to add any final comments on the topics covered by this survey or the survey itself, you may do so here (optional): ____

Please indicate below, if you agree that your survey entries are used for research purposes (the dissertation on academic air travel reduction and virtual communication):
☐ Yes, my survey entries may be used for research purposes and as feedback to conference organisers.
☐ No, I wish that my survey entries are solely used for feedback to conference organisers and not for research purposes.
